# Significance of a social mobilization intervention for engaging communities in polio vaccination campaigns: Evidence from CORE Group Polio Project, Uttar Pradesh, India

**DOI:** 10.7189/jogh.11.07011

**Published:** 2021-03-10

**Authors:** Manojkumar Choudhary, Roma Solomon, Jitendra Awale, Rina Dey, Jagajeet Prasad Singh, William Weiss

**Affiliations:** 1 CORE Group Polio Project, Gurgaon, Haryana, India; 2 Indian Institute of Health Management Research (IIHMR) University, Jaipur, Rajasthan, India; 3 Department of International Health, Johns Hopkins Bloomberg School of Public Health, Baltimore, Maryland, USA

## Abstract

**Background:**

Globally, community engagement is an integral part of most public health programs and the social mobilization (SM) intervention of India’s polio eradication program is one such example that contributed to eliminating polio from the country. CORE Group Polio Project (CGPP), a partner of Uttar Pradesh (U.P.) SM Network executed its activities through a network of social mobilizers called Community Mobilization Coordinators (CMCs). These were deployed in polio high risk areas to perform awareness generation and trust-building activities with communities and achieved high coverage of polio vaccination during Supplementary Immunization Activity campaigns (SIAs). This paper measures the extent and outcomes of CMC community engagement in SM interventions and polio SIAs.

**Methods:**

This study used secondary, cluster-level data from Management Information System of CGPP India, including 52 SIAs held between January 2008 to September 2017 in 56 blocks/polio planning units, covering 12 districts of U.P. We used five indicators that reflected community engagement in polio SIAs and constructed a Community Engagement Index (CEI). Further, we estimated the difference in the CEI between CMC and non-CMC areas, using Generalized Estimating Equations (GEE) and also estimated treatment effects through Difference-in-Differences (DID) method using STATA.

**Results:**

Overall, 78.6% (95% confidence interval (CI) = 78.3, 78.8) of families from the study area were engaged in the polio SIAs and the extent of community engagement increased over time. The mean CEI of entire study period in CMC areas (85.8%; 95% CI = 85.6, 86.0) was significantly higher (*P* < 0.001) than that of non-CMC areas (71.3%; 95% CI = 71.1, 71.5). Over time, the SM intervention led to at least 11 percentage points increase in the CEI of CMC areas with about 17% of this achievement attributable to CGPP India’s SM efforts.

**Conclusions:**

The study findings suggest that intensive social mobilization efforts can significantly increase the extent of community engagement. The community engagement learnings of polio SM Network may be useful to achieve the desired outcomes of public health programs such as the National Health Mission (NHM) of India, that serves communities for multiple health issues.

Globally, most of the public health programs apply community engagement strategies such as social mobilization (SM) and Social and Behavior Change Communication (SBCC), to help achieve their desired outcomes. Thoroughly designed community engagement interventions are effective in addressing various public health challenges such as low immunization, undernutrition and anemia, low use of family planning, HIV and AIDS, malaria and other infectious diseases [1-7]. The Social Mobilization Network (SM Net), a partner of India’s polio elimination initiative, focuses its efforts on community engagement for polio vaccination. The SM Net has implemented several innovations and it is well recognized for its contribution to eliminating polio from the country [8-12]. It deployed community-level workers called Community Mobilization Coordinators (CMCs) to engage communities for achieving high coverage of polio vaccination campaigns (Supplementary Immunization Activity campaigns or SIAs).

Most of the reviewed peer-reviewed literature on the role of SBCC or SM in polio SIAs describes the intervention and assess the outcomes of polio vaccination campaigns [11,13,14]. A few studies crudely measure the extent of community engagement, using proxy indicators [8,15]. However, we could not find published literature, particularly from India, that thoroughly quantifies community engagement in polio SIAs. This paper defines and measures the magnitude of community engagement in polio SIAs. Also, it assesses the contribution of an SM intervention on community engagement. Among the seven domains of Community Health Worker Performance Measurement Framework, suggested by Agarwal et al. [16], this study focuses on the ‘Community access’ domain, particularly the utilization of polio vaccination services.

## METHODS

*Polio SM Net in UP* – The polio SM Net of Uttar Pradesh (UP), India was established in 2003 and its constituents included UNICEF, CORE Group Polio Project (CGPP), Rotary, the Indian Government’s and the World Health Organization’s (WHO) National Polio Surveillance Project (NPSP) [6]. The UP SM Net implemented social mobilization activities using CMCs, who were supervised by Block Mobilization Coordinators (BMCs), who were in turn supervised by District Mobilization Coordinators (DMCs). Since its formation, the SM Net has supported polio eradication through the following activities: (a) identification of high-risk polio areas; and, (b) working with underserved communities to plan, implement, and monitor SM and other immunization-related activities. It developed SBCC and training materials and implemented its SM activities across designated CGPP and UNICEF areas [15]. The CMCs, frontline social mobilizers of SM Net, were deployed in selected polio high-risk areas, designated as ‘CMC areas’, to advocate for vaccination, The CMC areas were considered high-risk for polio because these areas experienced more resistance to polio vaccination and included more hard-to-reach populations than non-CMC areas.

*Polio SIAs and SM Net intervention* – Polio SIA operations in UP are almost uniform across the districts and it include two main types: (1) fixed-site, booth-based vaccination (In polio SIAs, fixed site or booth-based vaccination refers to a process of dispensing oral polio vaccine to eligible children at kiosks (booths) set up at fixed sites in a community. These booths are temporary and located at different places such as health facilities, educational institutions, residential premises and transit places like railway stations, bus stops); and, (2) house-to-house vaccination. In CMC areas, the SM Net functionaries engage communities before, during and after each polio SIA. Before each SIA, CMCs perform various awareness generation and trust-building activities such as the following: (a) interpersonal communication (one-to-one and one-to-group) with caregivers and family members of children in the SIA age-group; (b) meetings with local influencers; and, (c) children's rallies. DMCs and BMCs help the government to prepare for SIAs by participating in development of micro-plans and ensuring the availability of necessary logistics and supplies. Generally, polio SIAs begin on a Sunday with fixed polio vaccination booths for one day. CMCs involve school children in encouraging the community to bring children less than five years old to the booths for vaccination. Following the booth based vaccination, house-to-house vaccination phase begins where the CMCs accompany vaccinators who vaccinate eligible children not vaccinated at booths. If the team encounters refusal, CMCs engage the help of local influencers to try to convince resistant families to allow their children to be vaccinated. After completion of an SIA, the SM Net functionaries visit all the houses with unvaccinated children and encourage them to go for polio vaccination in the upcoming/next SIA [9,17,18].

### Study design

This research followed a quasi-experimental design with a nonequivalent comparison group. Although the study areas (CMC and non-CMCs) belong to the same block (In India, sub-district is known as ‘block’ and it is the lowest governmental administrative unit) or polio planning unit (Polio planning units – are the smaller clusters of urban areas (urban wards) from an administrative block or a city [19]) of a district, they had dissimilar socio-demographic profiles. The CMC areas had a lower level of female literacy and consisted of more Muslim population than the non-CMC areas (Table S5 in the **Online Supplementary Document**). The control-group time-series design consisted of a series of observations and measured the dependent variable on 52 occasions for both the CMC and non-CMC areas.

*Data source –* We carried out a secondary analysis of data routinely collected through the CGPP India project Management Information System (MIS). Of interest of this paper, the CGPP MIS captures information about various activities and results surrounding each polio SIA [15,18].

### Statistical analysis

***SIAs and analysis period*** – The analysis covers 52 polio SIAs held from January 2008 to September 2017 in 56 blocks/polio planning units, covering 12 districts of Uttar Pradesh. For the purpose of this study, ‘January 2008’ is considered as the start point. Although data for earlier SIAs were available in the CGPP India MIS, prior to January 2008, the reporting laid more emphasis on qualitative rather than quantitative data. We selected ‘September 2017’ as the endpoint of the study because, after this time, CGPP India altered its approach in some of the areas by introducing a low-intensity SM Net intervention without CMCs, intervening only through block-level functionaries.

We have not performed any sampling or sample size determination procedure and included all the 56 geographic areas (ie, blocks/polio-planning units) where CGPP had its SM intervention during the study period (ie, from January 2008 to September 2017). Similarly, we included all the 52 SIAs that had a complete operation (booth-based and house-to-house vaccination) and covered all the geographic areas. Note also that 25 SIAs (out of 77) held during the study period were excluded from the analysis because these SIAs had either partial operations (ie, the SIAs that included only one of the two main types of operations) or incomplete geographic coverage. Also, we excluded two CGPP blocks (out of 58) that were not covered at the start of the study period (Table S1 in the **Online Supplementary Document**). Inclusion of SIAs with the complete operation and geographic areas covered for the entire study period allowed us to measure the facts (eg, outcomes of polio SIAs) appropriately and avoided the misrepresentation and over- or under- estimation of SIA outcomes.

***Dependent variable (CEI)*** – At first, we computed indicator variables to quantify the performance of both the fixed-site and house-to-house vaccination activities of polio SIAs. Then we computed a Community Engagement Index (CEI) of polio SIAs (ie, the dependent variable), a composite indicator that quantifies the extent of community engagement, incorporating both the two major components of SIA operations.

***Construction of a Community Engagement Index (CEI) of polio SIAs*** – The concept of community engagement is complex, but it reflects the empowerment of communities, community leaders and community organizations to achieve the desired goals of different initiatives [20]. We considered community engagement in polio SIAs as the involvement and support of community members, particularly of family members of eligible children, in the polio vaccination drives. We followed the guidelines and recommendations specified in the ‘Handbook on Constructing Composite Indicators: Methodology and User Guide’ and allied literature that recommends or used this handbook [21-25]. In specific, we performed the recommended ten steps [21], while constructing the ‘CEI of polio SIAs’ (See **Box 2**).

Box 2 **Ten Steps of Computing Community Engagement Index.**

***1. Developing a theoretical framework*** – Using the operational definition of community engagement in polio SIAs (**Box 1**), we attempted to develop a theoretical framework for community engagement in polio SIAs by describing the components of polio vaccination campaigns in the study area.

*2.* ***Selection of variables*** *–* Following the theoretical framework, we considered the variables (ie, five outcomes/performance indicators of polio SIAs) with a similar measurement scale and appropriately represent the components of polio SIAs.

3. ***Data treatment/Imputation of missing data*** *–* The presence of outliers was checked and the unjustifiable extreme values were adjusted with the average (mean) values of each of the indicators. We used a single imputation method and replaced the missing data with the average (mean) values of the immediately available one-year period data (ie, the data of 5 SIAs, conducted from October 2012 to September 2013). We separately imputed the missing data for each of the geographical unit (ie, block/polio-planning unit) from intervention and non-intervention areas, for both the indicators. Then, we reversed the form of negatively coded variables to positive items.

4. ***Multivariate analysis*** – The CGPP database provided enough number of cases to perform Principle Components Analysis (PCA) and follow the below specified two rules of thumb specified in the “Handbook on constructing composite indicators…” for performing PCA [21].

• Rule of 10 – There are more than 10 cases for each variable

• Rule of 5:1 ratio – The cases-to-variables ratio is more than 5

We applied Principle Components Analysis (PCA) to identify groups of indicators, using SPSS software. In PCA, we considered eigenvalues above ‘1’ to determine the number of factors/components. In the PCA model, we used the different rotation methods to check for correlation among the items of each of the components. We used a recommended cut of correlation value greater than ‘0.32’ of less than ‘-0.32’ to identify components with a significant correlation among the items. Also, we attempted to identify complex items in the PCA model by observing the individual items with factor loading greater than ‘0.30’ for two or more components. We used Cronbach Coefficient Alpha to measure the internal consistency in the set of individual indicators, ie, how well the indicators describe a unidimensional construct. This helped in identifying sub-groups of indicators or groups of study blocks/polio-planning units that are statistically similar. We considered ‘0.6’ as a cut-off value as a reliability threshold. Further, we used Mahalanobis distance (using SPSS) to identify cases that are multivariate outliers, ie, a combination of unusual scores on at least two variables.

5. ***Normalization of data*** – As the variables under consideration, ie, the five outcome indicators have different measurement scales (ie, multipliers ranged from 100 to 10 000), we used the ‘Min-Max’ method among the different recommended methods to normalize the data. All the five indicators were converted to individual indices using the following formula:

Dimension index = (Actual value of an indicator – Minimum expected value of an indicator) / (Maximum expected value – Minimum expected value of an indicator)

*6.* ***Weighting and aggregation*** – After computing individual indices (with values ranging from ‘0’ to ‘1’) for all five indicators, we computed sub-indices for all three PCA components and final, community engagement index, using different aggregation methods. At the first level, we created an aggregated index by giving equal weight to all three PCA-based indices. The second type of index considered, ‘0.5’ weight to both the two major dimensions, ie, 1) Booth-based performance and 2) House-to-house vaccination. The next category included factor loading (of PCA analysis) and created a weighted index. While choosing components/factors and giving PCA-based weights, we followed the below-mentioned three standard practices.

(i) factors that have associated eigenvalues larger than one

(ii) factors that contribute individually to the explanation of overall variance by more than 10%

(iii) PCA components contribute cumulatively to the explanation of the overall variance by more than 60%.

7. ***Robustness and sensitivity analysis*** - We gauged the robustness of the composite indicator by performing uncertainty and sensitivity analysis. Specifically, we attempted to address below specified potential sources of uncertainty while computing CEI of polio SIAs:

• *Selection of indicators* – included all the indicators that can be theoretically linked to the index.

• *Normalization* - normalized all indicators before weighting and aggregation.

• *Weighting and aggregation method* – used different weighting schemes and different aggregation methods to check for the uncertainty.

**8. *Back to the real data* –** We checked the correlation of computed CEI with the individual variables and assessed whether or not the composite indicator (ie, CEI) is overly dominated by a few indicators. Also we explained the relative importance of the sub-components of the composite indicator.

**9. *Link to other variables*** – As our literature search could not find a similar composite indicator that assesses community engagement, we have not performed any analysis to link the CEI with published indicators.

**10. *Presentation and visualization*** – We visually presented the computed CEI in different ways visually, using a bar and spider/radar diagrams to interpret the results.

**Figure 1:**
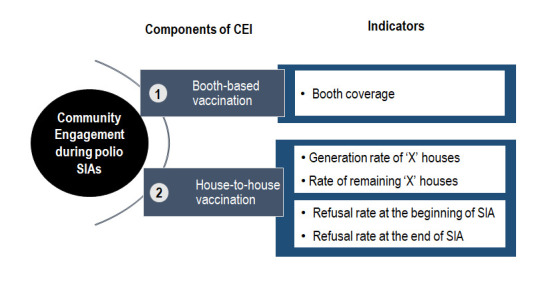
Theoretical framework of community engagement in polio SIAs.

***Theoretical framework to construct CEI*** – Community engagement in the polio SIAs is an ongoing process and it is not limited to vaccination days only [26]. The government of India and other stakeholders of the polio eradication initiative (including polio SM Net partners) attempt to engage communities at every stage of polio SIAs, ie, pre- (preparatory phase), during- (execution phase) and post-campaign (assessment or follow-up phase). Ideally, a composite measure of community engagement should include the indicators related to all the three phases of an SIA (ie, pre-, during- and post-campaign) and community processes. However, the unavailability of reliable data for the pre- and post-campaign phases, restricted us to consider only the selected indicators of polio campaigns. **Figure 1** depicts the theoretical framework of community engagement in polio SIAs. The two components (vaccination activities of SIAs, ie, [a] booth-based and [b] house-to-house vaccination) of polio SIAs are considered as two dimensions of community engagement. The framework also specifies the indicators against each component.

***Selection of variables for CEI**** –* Among the various indicators quantifying success in community engagement, we selected the following five to compute the CEI of polio SIAs:

1. *Booth coverage* – The percentage of eligible children vaccinated at the polio SIA booths (fixed site vaccination). The denominator of this indicator includes the total number of children vaccinated in the previous polio campaign.
2. *Rate of ‘X’ houses generated during an SIA* – The percentage of ‘X’ houses (ie, the households where an unvaccinated child is present or the vaccinators do not know the SIA vaccination status of all children) generated during the house-to-house activity of an SIA. The total number of houses visited by house-to-house vaccination teams of an SIA is the denominator of this indicator. Whereas, the numerator includes the ‘number of ‘X’ houses marked at the beginning phase (ie, the first house visit that usually happens during Day 2 to Day 6 of an SIA) of house-to-house vaccination activity.
3. *Rate of remaining ‘X’ houses at the end of an SIA* – This is a percentage of remaining ‘X’ houses at the end of the house-to-house activities of an SIA. The total number of houses visited by house-to-house vaccination teams of an SIA is the denominator of this indicator.
4. *Refusal rate at the beginning phase of house-to-house vaccination of an SIA* – This is the number of households who refused polio vaccination at the beginning phase (ie, first visit) of a house-to-house activity of an SIA (Marked as ‘XR houses’ in the vaccinators’ tally sheet) against every 10 000 households visited by house-to-house vaccination teams.
5. *Refusal rate at the end of an SIA* – This is the number of households who refused polio vaccination at the end of house-to-house activity of an SIA (Marked as remaining ‘XR houses’ in the vaccinators’ tally sheet) against every 10,000 households visited by house-to-house vaccination teams.

These indicators are proxies for success in engaging the community, representing the collaborative efforts of different stakeholders of the polio eradication initiative (see operational definition of community engagement in **Box 1**). However, they also reflect supplies/logistics, micro-planning, surveillance and government vaccinators’ efforts. Out of five, one indicator (ie, Booth coverage) indicates the community’s engagement during the fixed-site (booth-based) vaccination. In contrast, the other four indicators show the community’s engagement during the house-to-house vaccination of polio campaigns (The second component of CEI). The mean values of all five positively coded/scored indicators used for the computation of CEI are provided in Table S2 in the **Online Supplementary Document**.

Box 1 **Operational definition of Community Engagement Index (CEI) of polio SIAs.**

Following the definition of community engagement of the Centers for Disease Control and Prevention (CDC) [27], this study endeavored to operationally define the ‘Community Engagement in Polio SIAs’ as: *‘Collaborative efforts of Government of India (ie, Ministry of Health and Family Welfare), development organizations (including WHO/NPSP, Rotary and SM Net partners, ie, UNICEF and CGPP), community-based institutions (eg, academic institutions, religious institutions, professional institutions, etc.) and community members (including influencers) to achieve the high level of polio SIA vaccination.’*

***Components of CEI derived through multivariate analysis*** – The PCA analysis extracted three components with eigenvalues above ‘1’ (See Figure S1 in the **Online Supplementary Document**). The first component included a single item, whereas the other two components included two items. All the five items had a high level of factor loading and the PCA model explained about 85 percent of the variance. Reliability statistics revealed that the items were internally consistent (Cronbach’s alpha >0.6) in both the two components that included two indicators (See Table S3 in the **Online Supplementary Document****).** Although an analysis, using the Mahalanobis distance method, found about 1.9 percent of total 5824 records as multivariate outliers, we have not excluded or modified the values of these outliers and used their original values for further analysis.

The normalized mean values (ranging from 0 to 1) of all five indicators and three components derived through PCA are presented in Table S4 in the **Online Supplementary Document**. We looked at different weighting schemes and picked a scheme with moderately estimated values of CEI. Among the four ways we tried to compute the CEI, we preferred to use the CEI that is based on a 50-50 approach (giving equal weights to booth-based and house-to-house vaccination) and follows the theoretical framework for further analysis. This method yielded moderate estimates against the other three weighting methods (See Table S6 in the **Online Supplementary Document**).

The CEI was computed separately for CMC and non-CMC areas from each block and every SIA. After calculating CEI, we performed descriptive analysis – computed an aggregated average (mean) value (including all blocks and SIAs) of the entire study period for the CMC and non-CMC areas, separately.

After conducting the exploratory analysis (See Appendix S1 in the **Online Supplementary Document**), we performed the following analysis suited for the nature of data (ie, clustered and nested data).

***Generalized Estimating Equations (GEE) analysis*** – We assessed the difference between CEI of CMC and non-CMC areas, using GEE - based analysis in STATA. Similar to Weiss et al. (2011), we performed GEE analysis that accounted for the longitudinal/panel nature of the data including block/polio planning area level Intra-cluster correlation (ICC). We used ‘Quasi-likelihood under the independence model criterion (QIC)’ as the model selection method [15]. The GEE model with the lowest QIC was considered as the most appropriate one among the other competing models with different correlation structures, eg, exchangeable, auto-regressive, unstructured etc. (See Appendix S1 in the **Online Supplementary Document** for details).

***Difference-in-Differences (DID) analysis*** – We compared the differences between the CEI values of presumed ‘Baseline’ and ‘Endline’ of the study. For this, we used ‘diff’ command in STATA (IBM, Armonk, NY, USA), developed by Villa [28] and estimated Difference-in-Differences (DID) treatment effects, using unadjusted, adjusted and kernel PSM methods (See Appendix S1 in the **Online Supplementary Document** for details). The DID analysis is widely used to assess the impact of an intervention using a panel or repeated cross-sectional data [28]. In the analysis, possible covariates included selected characteristics of CMC and non-CMC area, ie, level of urbanization, female literacy rate, percent Hindu/Muslim population, and average household size (Total individuals in a household) (See Table S5 in the **Online Supplementary Document**).

## RESULTS

### Extent of community engagement in polio vaccination campaigns

**Figure 2:**
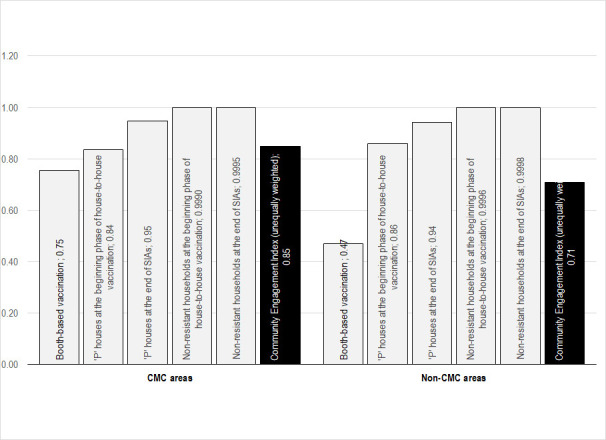
Mean values of individual indices and unequally weighted Community Engagement Index by CMC-level intervention status, January 2008 to September 2017.

As mentioned before, this study measures the extent of community engagement in polio SIAs through a CEI, a composite indicator computed on the five indicators. **Figure 2** presents the mean values of individual indicators along with CEI for the CMC and non-CMC areas separately. The mean values vary by indicators for both areas. Among the five, the indicators related to acceptance of polio vaccination (The last two indicators associated with the proportion of non-resistant households) have higher values. In contrast, the first indicator, ie, booth-based vaccination, has the smallest values. All five indices are reflected in the CEI and CEI values fall in between the five individual indices. The CEI values ranged from 0 to 1. The zero CEI value indicates no engagement of communities.

**Figure 3:**
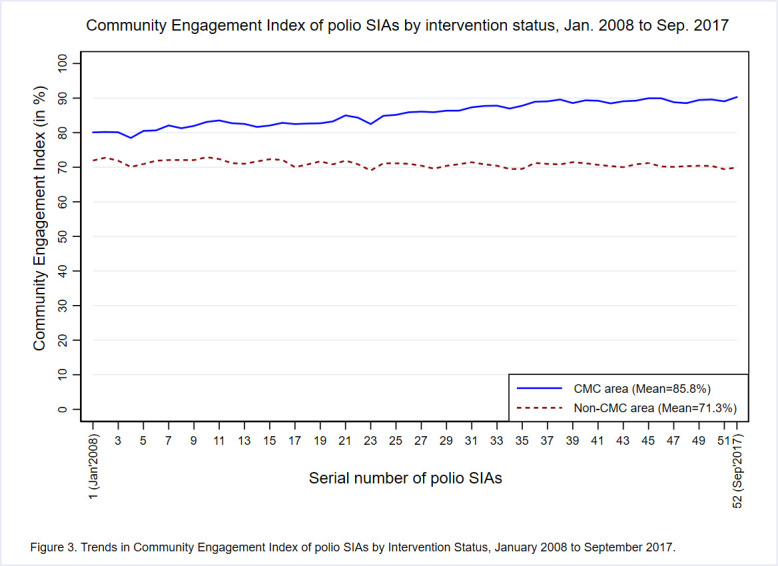
Trends in Community Engagement Index of polio SIAs by Intervention Status, January 2008 to September 2017. Each line represents the mean value for Community Engagement Index (CEI, presented as percentage of families that were engaged in the polio SIAs) of polio SIAs, during the entire study period. The mean value is calculated at the block level separately for CMC and non-CMC areas. The blue line and broken brown line represents to the CMC and non-CMC areas, respectively.

Overall, three-fourths (mean = 78.6%; 95% CI = 78.3, 78.8) of families from the entire study area (including CMC and non-CMC areas) were engaged in polio SIAs. **Figure 3** provides trends in the extent of community engagement in polio SIAs held from January 2008 to September 2017 by study area. In the unadjusted analysis, CMC areas had a significantly higher (*P* < 0.001) mean CEI (85.8%; 95% CI = 85.6, 86.0) than non-CMC areas (71.3%; 95% CI = 71.1, 71.5), across the entire study period. The difference in the mean CEI between CMC and non-CMC areas was smaller in the earlier SIAs and continued to increase over time in favor of the CMC areas. It appears that there was a substantial increase in the CEI in CMC areas over time, whereas the CEI trend in non-CMC areas was relatively static. The gradual increase in CEI of CMC areas resulted in a widened gap of 14.5 percentage points between the CEI of CMC and non-CMC areas. The difference in the CEI between CMC and non-CMC areas varied by district, with the largest gap observed in Sitapur District (See Figure S2 in the **Online Supplementary Document**).

### Longitudinal analysis of community engagement of CMC and non-CMC areas

**Table 1** presents the results of GEE-based, bivariate and multivariate analysis, along with the mean values of the CEI for the entire study period (ie, from January 2008 to September 2017). The difference in CEI between the CMC and non-CMC areas varies across districts, place of residence and time of year. The bivariate analysis detected a significant difference (*P* < 0.01) between the CMC and non-CMC areas for all independent variables. However, the CEI did not significantly differ (*P* = 0.684) by ‘Place of Residence’ in the initial multivariate analysis and hence this variable was excluded from the final multivariate models. The multivariate analysis (that adjusted for the correlation of data within study districts over time) found that the mean CEI was statistically significantly higher (*P* < 0.001) in CMC areas as compared to non-CMC areas. This finding was also consistent for all districts and all SIAs in the study period (*P* < 0.01).

**Table 1:** Mean level of community engagement in polio SIAs by district, place of residence, SIA month and intervention status, January 2008 to September 2017

By:	Community Engagement Index (in %) (95% Confidence Interval)		
**CMC Areas (n [blocks] = 56; Obs. per block = 52)**	**Non-CMC Areas (n [blocks] = 56; Obs. per block = 52)**	***P*-value**
**Overall**	85.8 (85.6, 86.0)	71.3 (71.1, 71.5)	<0.001*†
**District:**			
Baghpat	86.3 (85.9, 86.6)	72.2 (71.8, 72.6)	<0.001†
Bareilly	84.5 (83.7, 85.3)	72.1 (71.7, 72.4)	<0.001†
Mau	86.4 (85.3, 87.5)	67.4 (67.1, 67.7)	<0.001†
Meerut	86.7 (86.3, 87.2)	78.3 (77.7, 78.9)	<0.001†
Moradabad	84.7 (84.3, 85.1)	73.9 (73.4, 74.4)	<0.001†
Muzaffarnagar	85.9 (85.3, 86.5)	70.1 (69.7, 70.5)	<0.001†
Rampur	86.9 (86.2, 87.5)	72.1 (71.7, 72.5)	<0.001†
Saharanpur	87.6 (86.9, 88.2)	75.2 (74.7, 75.7)	<0.001†
Sambhal	84.0 (83.6, 84.5)	75.6 (75.0, 76.2)	<0.001†
Shahjahanpur	87.2 (86.7, 87.8)	69.5 (69.0, 70.0)	<0.001†
Shamli	85.4 (84.7, 86.2)	70.5 (70.0, 71.0)	<0.001†
Sitapur	84.9 (84.3, 85.5)	60.3 (60.0, 60.6)	<0.001†
**Place of residence:**			
Rural	86.0 (85.8, 86.2)	71.1 (70.9, 71.3)	<0.001*‡
Urban	84.2 (83.7, 84.7)	72.7 (72.2, 73.3)	<0.001*‡
**Time of year:**			
January to March	86.0 (85.7, 86.2)	71.8 (71.4, 72.1)	<0.001†
April to June	85.6 (85.2, 86.0)	70.5 (70.1, 70.9)	<0.001†
July to September	86.4 (85.9, 86.8)	70.9 (70.4, 71.4)	<0.001†
October to December	85.3 (84.9, 85.7)	71.7 (71.1, 72.2)	<0.001†

SIA – supplementary immunization activity, CMC – community mobilization coordinator

*Bivariate analysis of Community Engagement Index by Intervention Status using generalized estimating equations (standard errors adjusted for clustering by block/Intervention Status)

†Statistically significant difference (*P* < 0.01) in multivariate analysis of CEI by Intervention Status controlling for District, and Time of year, and including interaction of District with Intervention Status, using generalized estimating equations (standard errors adjusted for clustering by block/Intervention Status). Multivariate GEE models included an ‘exchangeable’ correlation matrix. QIC of overall GEE model was 171003.2

‡Not included in the multivariate GEE analyses, as the initial analysis (ie, the model included: Community Engagement Index, Intervention status and Place of residence) found an insignificant difference (*P* = 0.684) between the Community Engagement Index of rural and urban areas.

Results of Difference-in-Differences (DID) method based analysis presented in **Table 2** show that the CGPP’s SM intervention led to about 11 percentage points increase in the extent of community engagement from CMC areas. In the DID analysis, we presumed the first five (ie, serial No. 1 to 5) and the last five (ie, serial No. 48 to 52) SIAs of the study period as Baseline and Endline, respectively. Unadjusted DID treatment-effects based estimate shows a significant increase (*P* < 0.001) of 11.7 percentage points in the CEI from CMC areas. Adjusted DID treatment-effects (Including two significant covariates, ie, percent Hindu population and average household size) explaining more variance (R-square = 0.72) found similar effects of 11.7 percent points. Similarly, the Kernel PSM method estimated DID treatment effects of 11.3 percent points. The initial Kernel PSM model included three covariates: female literacy rate, percent Hindu population and average household size. Out of three, one variable (ie, average household size) that was not achieving balance after matching was removed from the PSM model. The Kernel PSM model reduced the covariate imbalance (ie, bias associated with the characteristics of CMC and non-CMC areas) from 110.5 percent to 1.5 percent (See Table S7 and Figure S3 in the **Online Supplementary Document**).

**Table 2:** DID treatment effects of CGPP’s SM Net intervention on the extent of community engagement in polio SIAs of CMC area – a comparison between presumed Baseline (January 2008 to June 2008) and Endline period (September 2016 to September 2017)

Difference between the CEI of CMC and non-CMC area		Estimation method		
**Unadjusted**	Adjusted*	**Kernel propensity-score matching†**
Presumed baseline period (Initial 5 SIAs of the study period, ie, Jan. to Jun. 2008)	CEI of non-CMC area	71.777	68.104	72.629
	CEI of CMC area	79.971	73.726	79.971
	% points difference	8.194	5.622	7.342
	Std. Err.	0.426‡	0.532‡	0.372
	*P*-value	<0.001	<0.001	<0.001
Presumed Endline period (Last 5 SIAs of the study period, ie, Sep. 2016 to Sep.)	CEI of non-CMC area	70.316	66.643	71.626
	CEI of CMC area	90.270	84.026	90.270
	% points difference	19.954	17.383	18.644
	Std. Err.	0.337‡	0.657‡	0.372
	*P*-value	<0.001	<0.001	<0.001
Difference-in-differences (DID)	% points difference	11.760	11.760	11.303
	Std. Err.	0.577	0.619	0.527
	*P*-value	<0.001	<0.001	<0.001
	R-square	0.71	0.72	0.78

CEI – community engagement index, CMC – community mobilization coordinator, SIA –supplementary immunization activity, DID – difference-in-difference, Std. Err. – standard error

*Adjusted for the effects of two covariates (percent Hindu population and average household size).

**†**Adjusted for the effects of two covariates: female literacy rate and percent Hindu population (No interaction term was used); matching reduced mean bias to 1.5%, from an unmatched mean bias of 110.5%.

‡ Bootstrapped standard errors (50 replications).

## DISCUSSION

The community-level SM intervention of CGPP in its catchment areas has significantly contributed to engaging communities in polio SIAs. We believe that the gradual increase of 11 percentage points in the CEI of CMC areas made a great difference in engaging communities and building the herd immunity, as most of the CMC areas had pockets of resistance to polio vaccination. Over time, increased CEI reflects an increase in self-motivation among communities for polio SIA vaccination, as the ‘Booth coverage’ indicator weighs heavily in the calculation of the CEI. The ‘Booth coverage’ quantifies an important sense of community engagement, ie, the proportion of community members who themselves were active in bringing their children to the SIA booths for vaccination. In contrast, the coverage of house-to-house vaccination does show the proportion of communities that were engaged but in a more passive way, accepting the offer of house-to-house vaccination teams. In order to engage families in upcoming SIAs, the profile of disengaged families, especially those not bringing children to booths, need to be studied. The study finding indicates that there was district-level clustering in CEI. Therefore, it is suggested that district planners need to thoroughly review the performance of polio SIAs, particularly ‘Booth coverage’ of non-CMC areas and carry out pre-campaign awareness generation and mobilization activities.

CMCs played a significant role in engaging and convincing communities, particularly mothers/caregivers and family members of eligible children, about the benefits of vaccinating their children for polio and other life-threatening vaccine-preventable diseases. CGPP India trained and built their capacities on convincing communities, using community-relevant communication materials and job aids. As mentioned before, the CMCs performed various awareness generation and trust-building activities to mobilize communities to take their children to polio booths or routine immunization sessions for vaccination. They keep records of all households and vaccination of eligible children and track the vaccination defaulters, including families resisting vaccination [18]. Also, their critical role was to ensure the childhood immunization of all eligible children, for which they often used local influencers to turn resistant families into acceptors of polio/routine immunization [17,29,30].

The study findings are somewhat similar to and supplement previous analyses that found the SM Net initiative contributed to increased levels of polio vaccination during SIAs [8,15]. Even though the areas designated for CMCs are more challenging for vaccination efforts, the level of community engagement in CMC areas was greater as compared to non-CMC areas. Using the concept of ‘percent change’ or ‘attributable risk’ and the below mentioned formula, one can estimate that about 17% of achieved level of community engagement in CMC areas can be attributed to the CGPP India’s SM efforts:

Attributable contribution of SM Net intervention in the achieved extent of community engagement in CMC areas ≈ [(Estimated extent of community engagement form CMC areas – Estimated extent of community engagement from non-CMC areas) / Estimated extent of community engagement from CMC areas] × 100.

In absolute numbers, out of the 546 314 targeted average households of polio SIAs, the SM efforts of CGPP alone engaged approximately 79 217 families in CMC areas during each polio SIA from January 2008 to September 2017. Number of households from CMC areas that were engaged in each SIA through CGPP India’s SM efforts is estimated as:

(Total number of targeted households in CMC areas × Proportion of households from CMC areas that were engaged in each polio SIA) × Proportion of engaged community from CMC areas attributable to CGPP’s SM efforts.

Further research can use the different techniques of estimating treatment effects and assess the effectiveness of SM intervention in engaging communities during the different phases of the polio eradication initiative (eg, polio-endemic, polio-non-endemic period). Additionally, the key SM activities and other factors that determine the magnitude of community engagement can be identified. The adaptability of the SM Net approach to other public health issues can also be tested.

### Limitations

Similar to the previously conducted study by Weiss et al. [15], this study also has a limitation about the degree of comparability between the CMC and non-CMC areas. The study areas, particularly the CMC areas, were not randomly assigned and included the polio high-risk areas where a high proportion of communities were not accepting the polio vaccination. The actual difference between the extent of CEI might have been more than estimated. Also, the unavailability of complete quantitative data for the beginning period of SM intervention (ie, from 2003 to 2008) is another limitation in assessing the actual treatment effects that forced us to assume data of initial SIAs as ‘Baseline’ for this study.

Principally, community engagement is considered as one of the programmatic approaches/elements to improve the outcomes of any intervention/program. It is a collaborative process that involves groups of individuals or organizations to address any issue affecting the people [20,27]. Although the polio elimination efforts in the study areas attempted to engage communities before, during and after the polio SIAs, this research used outcome or coverage indicators of polio SIAs and computed a CEI that quantifies the extent of community engagement in polio SIAs. The CEI is a composite indicator based on standardized values of selected five indicators representing both the booth-based and house-to-house vaccination approaches.

The CEI should not be interpreted as a single measure of the ‘performance of polio SIAs’ or effectiveness of polio SIAs. The overall performance of SIAs, measured through another indicator ‘Percentage of eligible children vaccinated during SIA’ hovered around 100 percent. However, this indicator is based on the proxy denominator, ie, ‘Number of children vaccinated in the previous SIA’ that depends on the performance of previous SIA and it may not include the accurate number of total eligible children. In contrast, the CEI shows the extent of communities that were engaged during both the booth-based and house-to-house vaccination. Although the refusal rate is one of the parts of Rate of remaining ‘X’ houses, this study purposively included ‘Refusal’ related two indicators, while constructing the CEI. It was essential to add the extent of resistance to polio vaccination, as there were many instances of one refusal family that led to many refusals, even an entire community [31]. In the study area, the community member had many myths and misconceptions and many rumors were prevailing in the study areas that led to resistance in the population [29,32].

## CONCLUSIONS

Better community engagement performance of CMC areas suggests that intensive SM interventions are useful in attracting dis-engaged communities and achieve the desired outcomes. Any public health program that faces the challenges of resistance or low acceptance can employ the community engagement strategies and approaches similar to the Polio SM Net initiative. The SM/SBCC tools and techniques developed by the SM Net initiative [9,29,30,33] can be applied to other public health programs for encouraging communities to adopt the desired behaviors. The SM Net approach can also be used in programs that serve communities in multiple health issues like India’s National Health Mission (NHM). For instance, the NHM or Integrated Child Development Scheme (ICDS) of India can adopt the community engagement and capacity building strategies of polio SM Net and build communication skills as well as the micro-planning capacities of their frontline workers (ie, Accredited Social Health Activities (ASHAs) of the NHM and Anganwadi workers of ICDS).

## Additional material

Online Supplementary Document
