# Supplementary material for: Significance of a social mobilization intervention for engaging communities in polio vaccination campaigns: Evidence from CORE Group Polio Project, Uttar Pradesh, India
Source: J Glob Health. 2021 Mar 10;11:07011. doi: 10.7189/jogh.11.07011 (PMC7956133; doi:10.7189/jogh.11.07011)
Supplement: Online Supplementary Document [file jogh-11-07011-s001.pdf]

Appendix

Appendix Table S1. List of polio SIAs along with their geographic coverage and inclusion status in the study, January 2008 to September 2017

No.	Polio SIA dates	No. of CGPP districts covered	No. of CGPP blocks/ polio planning units	SIA type	Inclusion/ Exclusion status	SIA No.
1	06 January 2008	12	56	NID	Included	1
2	10 February 2008	12	56	NID	Included	2
3	30 March 2008	12	56	SNID	Included	3
4	27 April 2008	12	56	SNID	Included	4
5	01 June 2008	12	56	SNID	Included	5
6	29 June 2008	12	56	SNID	Included	6
7	27 July 2008	05	24	SNID	Excluded	
8	03 August 2008	04	20	SNID	Excluded	
9	26 August 2008	05	24	SNID	Excluded	
10	14 September 2008	12	56	SNID	Included	7
11	18 October 2008	08	40	SNID	Excluded	
12	16 November 2008	12	56	SNID	Included	8
13	21 December 2008	12	56	NID	Included	9
14	01 February 2009	12	56	NID	Included	10
15	01 March 2009	12	56	SNID	Included	11
16	05 April 2009	09	36	Mop Up	Excluded	
17	21 April 2009	01	06	SNID	Excluded	
18	17 May 2009	09	46	SNID	Excluded	
19	05 July 2009	12	56	SNID	Included	12
20	26 July 2009	04	19	SNID	Excluded	
21	09 August 2009	12	56	SNID	Included	13
22	10 September 2009	12	56	SNID	Excluded (No booth day activity)	
23	04 October 2009	10	50	Mop Up	Excluded	
24	08 November 2009	12	56	SNID	Included	14
25	06 December 2009	10	50	SNID	Excluded	
26	10 January 2010	12	56	NID	Included	15
27	07 February 2010	12	56	NID	Included	16
28	21 March 2010	10	50	SNID	Excluded	
29	25 April 2010	10	50	SNID	Excluded	
30	23 May 2010	12	56	SNID	Included	17
31	20 June 2010	09	46	SNID	Excluded	
32	18 July 2010	01	04	SNID	Excluded	
33	19 September 2010	12	58	SNID	Included	18
34	14 November 2010	12	58	SNID	Included	19
35	23 January 2011	12	58	NID	Included	20
36	27 February 2011	12	58	NID	Included	21
37	27 March 2011	12	58	SNID	Included	22
38	24 April 2011	10	50	SNID	Excluded	
39	22 May 2011	10	50	SNID	Excluded	
40	26 June 2011	12	58	SNID	Included	23
41	26 July 2011	01	10	Mop Up	Excluded	
42	21 August 2011	10	50	SNID	Excluded	

No.	Polio SIA dates	No. of CGPP districts covered	No. of CGPP blocks/ polio planning units	SIA type	Inclusion/ Exclusion status	SIA No.
43	25 September 2011	12	58	SNID	Included	24
44	13 November 2011	08	40	SNID	Excluded	
45	15 January 2012	10	50	SNID	Excluded	
46	5 & 19 February 2012	12	58	NID	Included	25
47	18 March 2012	12	58	SNID	Included	26
48	15 April 2012	12	58	NID	Included	27
49	17 June 2012	12	58	SNID	Included	28
50	09 September 2012	12	58	SNID	Included	29
51	04 November 2012	12	58	SNID	Included	30
52	20 January 2013	12	58	NID	Included	31
53	24 February 2013	12	58	NID	Included	32
54	07 April 2013	12	58	SNID	Included	33
55	16 June 2013	12	58	SNID	Included	34
56	15 September 2013	12	58	SNID	Excluded (No booth day activity)	
57	24 November 2013	12	58	SNID	Included	35
58	19 January 2014	12	58	NID	Included	36
59	23 February 2014	12	58	NID	Included	37
60	24 April 2014	10	48	SNID	Excluded	
61	14 September 2014	12	58	SNID	Included	38
62	16 November 2014	12	58	SNID	Included	39
63	18 January 2015	12	58	NID	Included	40
64	22 February 2015	12	58	NID	Included	41
65	26 April 2015	12	58	SNID	Included	42
66	21 June 2015 & 13 September 2015	12	58	SNID	Included	43
67	22 November 2015	12	58	SNID	Included	44
68	13 December 2015	04	20	SNID	Excluded	
69	17 January 2016	12	58	NID	Included	45
70	21 February 2016	12	58	NID	Included	46
71	16 April 2016	11	52	SNID	Excluded (No booth day activity)	
72	29 May 2016	12	58	SNID	Included	47
73	25 September 2016	12	58	SNID	Included	48
74	29 January 2017	12	58	NID	Included	49
75	02 April 2017	12	58	NID	Included	50
76	02 July 2017	12	58	SNID	Included	51
77	17 September 2017	12	58	SNID	Included	52
NID = National Immunization Day; SNID = Sub-National Immunization Day						

Appendix Table S2. Mean values of indicators (original or reversely coded) used in the computation of Community Engagement Index, January 2008 to September 2017

Indicators	CMC area		Non-CMC area	
	Mean	SD	Mean	SD
1. Percentage children vaccinated at SIA booths (original coding)	77.1	9.58	47.6	11.92
2. Percentage of households with all vaccinated children (i.e., 'P' houses) at the beginning phase of house-to-house vaccination activity of polio SIAs	83.9	4.05	85.8	3.33
3. Percentage of households with all vaccinated children (i.e., 'P' houses) at the end of SIA	94.6	2.36	94.0	2.23
4. Rate of non-resistant households (accepters) at the beginning phase of house-to-house vaccination of polio SIAs (per 10,000 households)	9990.41	16.68	9995.89	6.70
5. Rate of non-resistant households (accepters) at the end of polio SIAs (per 10,000 households)	9995.43	16.25	9998.10	4.88

SD = Standard Deviation

Appendix Table S3. Components of five indicators (items considered for computing CEI) derived through principal component analysis

Indicators under consideration	Item/ factor loading[1]	Labeling of components derived through PCA	Cronbach's alpha
1. Percentage children vaccinated at SIA booths	0.972	**Component 1 –** CE in booth-based vaccination	---
2. Percentage of households with all vaccinated children (i.e., 'P' houses) at the beginning phase of house-to-house vaccination activity of polio SIAs	0.878	**Component 2 –** CE during a house-to-house vaccination	0.638
3. Percentage of households with all vaccinated children (i.e., 'P' houses) at the end of SIA	0.871		
4. Rate of non-resistant households (accepters) at the beginning phase of house-to-house vaccination of polio SIAs (per 10,000 households)	0.881	**Component 3 –** Acceptance to polio vaccination	0.731
5. Rate of non-resistant households (accepters) at the end of polio SIAs (per 10,000 households)	0.893		

The model explains 84.5% of the variance
Number of components were determined through Eigenvalues >1

[1] **Item/factor loading** in factor analysis/principal component analysis shows correlation between an item/variable and a component/factor.

Appendix Table S4. Mean values of individual indicators and PCA components used for computation of CEI

Indicator	Mean values (normalized)		Components of PCA	Mean values	
	CMC area	Non-CMC area		CMC area	Non-CMC area
1. Children vaccinated at SIA booths	0.771	0.476	**1.** CE in booth-based vaccination	0.771	0.476
2. Households with all vaccinated children ('P' houses) at the beginning phase of house-to-house vaccination of SIAs	0.839	0.858	**2.** CE during house-to-house vaccination*	0.892	0.899
3. Households with all vaccinated children ('P' houses) at the end of SIAs	0.946	0.940			
4. Non-resistant households (accepters) at the beginning phase of house-to-house vaccination of SIAs	0.99904	0.9996	**3.** Acceptance to polio vaccination*	0.99929	0.9997
5. Non-resistant houses (accepters) at the end of polio SIA	0.99954	0.9999			

* Mean values of sub-dimension indices computed through equal weight method, i.e., allotting equal weights (0.5 to each) to both the sub-dimension indices

Appendix Table S5. Covariate imbalance across study groups prior to matching

Covariate	CMC area (mean)	Non-CMC area (mean)	Difference	t statistic	p-value
Percent urban population, 2011	28.03	33.71	5.68	0.9606	0.3389
Female literacy rate, 2011	45.28	53.90	8.62	4.4858	<0.001
Percent Hindu population, 2011	38.62	62.41	23.79	7.1088	<0.001
Percent Muslim population, 2011	60.84	36.04	-24.81	-7.5743	<0.001
Average household size	7.48	6.08	-1.40	-15.6662	<0.001

Data sources: CMC (intervention) area = Household surveys of CGPP India (29)
Non-CMC area = Census of India, 2011 (30,31)

Note - Percent Hindu population (with positive correlation with exposure variable) is preferred against the percent Muslims to represent the socio-cultural profile. A preliminary list of covariates for PSM analysis included three covariates: a) female literacy rate and b) percent Hindu Population and c) average household size.

Appendix Table S6. Mean values of CEI computed through various computation methods

Aggregation method	Mean values of CEI	
	CMC area	Non-CMC area
Equal weights to all three PCA components and aggregation through arithmetic mean	0.8875	0.7918
Equal weights to all three PCA components and aggregation through Geometric mean	0.8809	0.7477
Unequal weights according to the theoretical framework (50-50 weights to two major components)	0.8583	0.7129
Weights according to factor loading of each of the indicators in PCA	0.8165	0.7618

Appendix Table S7. Difference in the mean of covariates after kernel matching (balancing test statistics of Kernel propensity-score matching model estimating DID treatment effects on community engagement in polio SIAs)

Variable	Treated (CMC area)	Control (Non-CMC area)	Difference	p-value
Female literacy rate	45.276	45.233	0.043	0.962
Percent Hindu population	38.618	39.052	-0.434	0.786

Appendix Figures

Screen plots

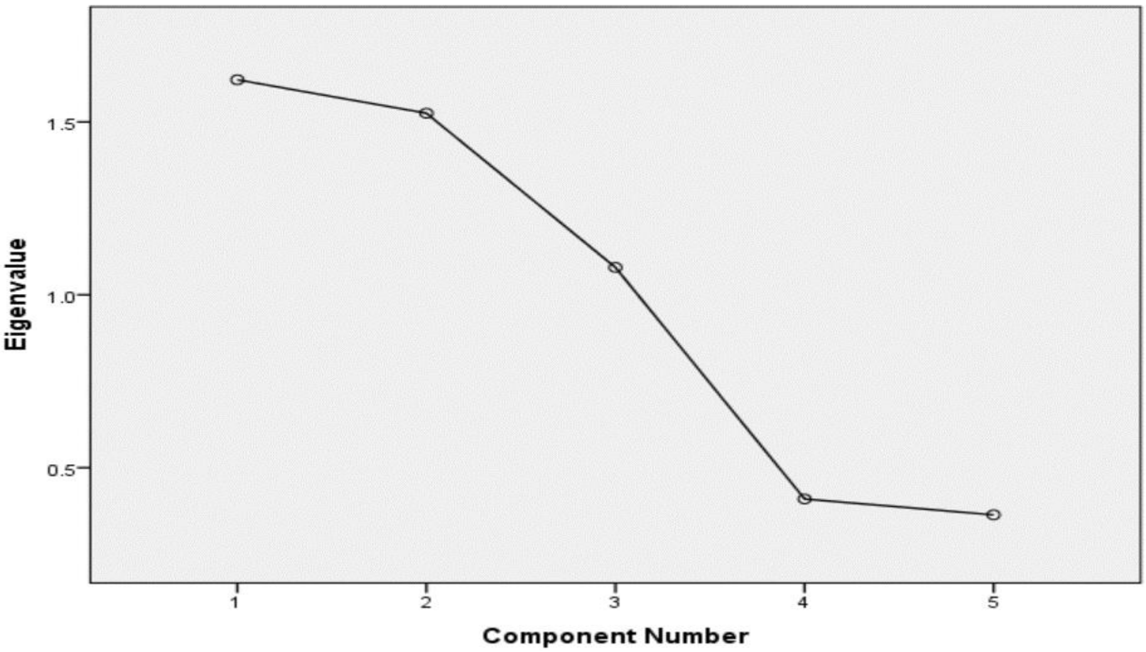

Appendix Figure S1. Screen plot of eigenvalues by components in the principal component analysis

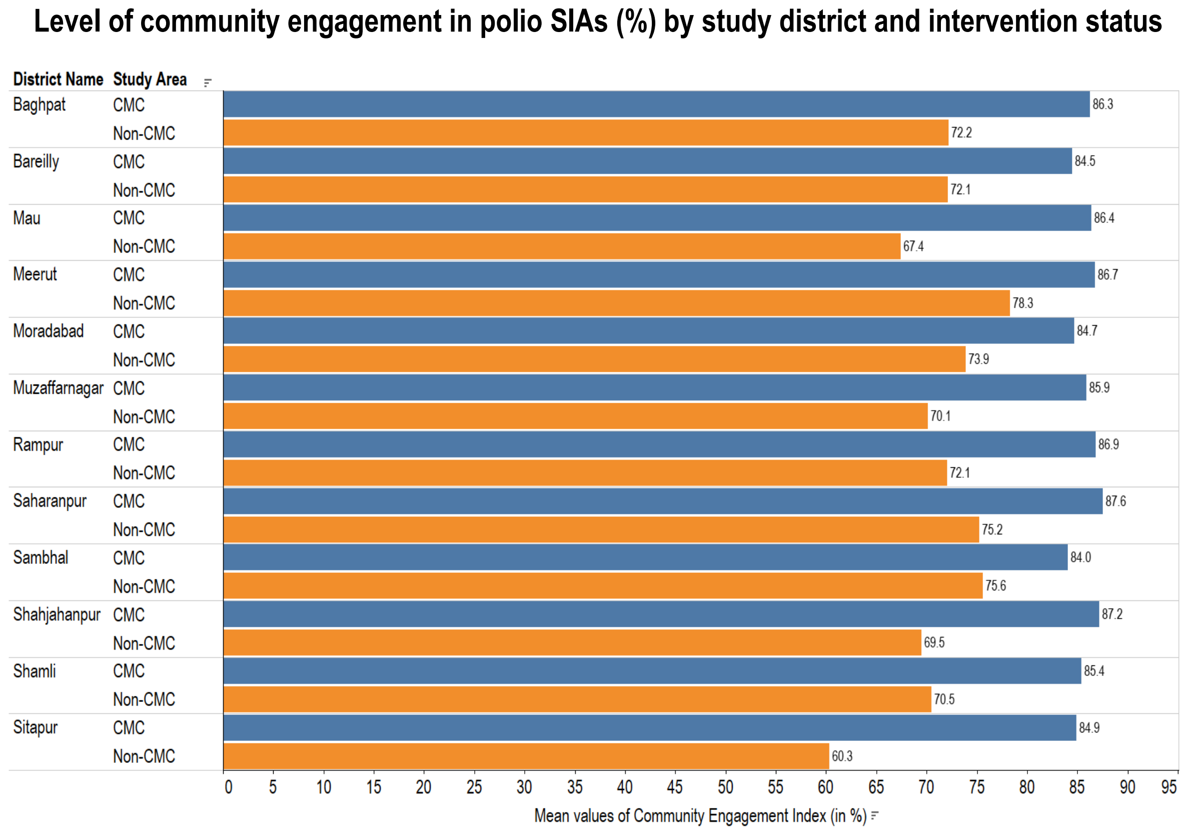

Appendix Figure S2. Mean level of community engagement in polio SIAs by District and Intervention Status, **January 2008 to September 2017.** Each bar in the graph represents the mean value for Community Engagement Index of polio SIAs. The mean value is calculated at the block level separately for CMC areas and non-CMC areas. The mean values from both the CMC and non-CMC areas are calculated separately for each district. The blue bars and orange bars represent CMC and non-CMC areas, respectively.

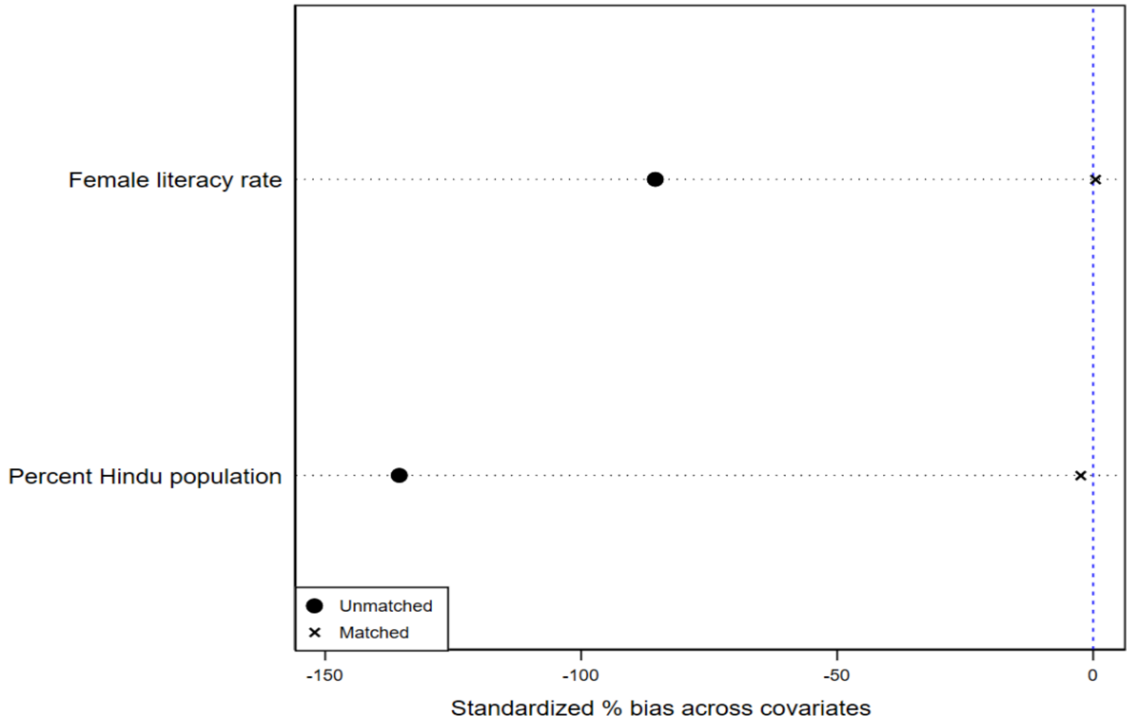

Appendix Figure S3. Diagnostic plots of Kernel PSM based analysis assessing before and after matching imbalance (mean bias) in the covariates of Community Engagement Index.
